# Supplementary material for: Components of the LINC and NPC complexes coordinately target and translocate a virus into the nucleus to promote infection
Source: PLoS Pathog. 2022 Sep 6;18(9):e1010824. doi: 10.1371/journal.ppat.1010824 (PMC9481172; doi:10.1371/journal.ppat.1010824)
Supplement: Attachment — Submitted filename: Spriggs et al. (Point-by-point Response).docx [file ppat.1010824.s001.docx]

**Reviewer #1:**

1. Abstract, Line 26: change to “for most DNA viruses…”

We have updated the text.

2. Figure 2 – Is the partial disassembly of the virus by treatment w/ DTT/EGTA established in the literature? Can you provide a reference on line 167?

SV40 undergoes partial disassembly in the ER where the VP1 inter-pentameric disulfide bonds are reduced by members of the PDI family of proteins. We use DTT to mimic this reaction because it is a reductant. We add the calcium chelator EGTA to replicate the subsequent decrease in calcium concentration that the virus encounters upon reaching the cytosol from the ER. This further disrupts the outer capsid shell by destabilizing VP1 inter-pentameric interactions. As requested, we have now included the appropriate references for this in the manuscript.

3. Figure 2 legend – D is not described. Maybe C on line 491 should be D? Also, the short and long exposure are not described.

We have corrected the figure legend.

4. The PCR analysis performed in Figure 2 is not described in the M&Ms and should be included.

We have added a description of the PCR analysis in the Materials and Methods section (line 735).

5. It’s unclear why Fig. 2E has significantly reduced FLAG-SR51-56 KASH in comparison to the deltaAD or in D? Please provide an explanation or consider adding quantitation of several replicates.

Although this is not entirely clear, one possibility is that the deltaAD construct is in a conformation that is more readily pulled down by the FLAG antibody when compared to the SR51-56 KASH construct. As requested, we have added a quantification from three independent replicates to the revised manuscript (line 509).

6. Is it possible that the non-infectious VP1 particles that enter the cell are intentional by-products of replication that have a pro-viral effect on SV40 infection or are the empty particles an effect of using high MOIs in these studies?

In these experiments, we added actinomycin D at the time of infection to inhibit virus transcription (and any subsequent replication) to ensure that any detected viral capsid proteins observed in the nucleus were derived from the incoming virus and not newly transcribed/translated viral capsids. We suspect that the non-infectious VP1 particles inside of the nucleus are the result of disassociated pentamers from the upstream BICD2-dependent disassembly. In physiological virus infection, these particles may be degraded at a rate that limits their arrival inside of the nucleus so it is certainly possible that our use of the high MOIs required to visualize SV40 inside the nucleus resulted in this phenotype. The entry of VP1 and VP3 (along with the viral genome) into the nucleoplasm does, however, raise the possibility that these capsids may have additional pro-viral effects post nuclear entry. Future studies may help to clarify this point.

7. Please include more detail on how the images and W blots were quantified in Fiji.

We have now included more detail on how images and western blots were quantified in Fiji (lines 691 and 760).

8. Figure 5G – The input Nup188 protein levels appear to be reduced in Nesprin-2-treated cells. Given that Nup188 interacts with Nesprin-2, is KD of Nesprin-2 reducing Nup188? Can this be quantified to demonstrate if input is not reduced or approached in another way possibly with an additional control KD such as RanBP2 KD? An explanation could be provided in the Discussion.

In the manuscript, we normalized VP1 to the IP’ed material and not the input, but we agree that the input NUP188 levels appear to decrease in the original Figure 5G. However, when these results were quantified (see right graph as requested), the data showed that while the NUP188 level was modestly reduced under Nesprin2 knockdown (~30%), this reduction is not statistically significant. We have therefore updated Figure 5G to include a more representative replicate.

**Reviewer #2:**

1. Line 280/281. If VP1 enters the nucleus independently of Nesprin-2 and VP3 (as shown in Figure 4D) then why is it described as in complex with VP3/genome? Seems it wouldn’t be in complex right? This was confusing.

We apologize for this confusion. We believe that a pool of VP1 still remains in complex with VP3/genome. Specifically, in the imaging data presented in Figure 3C, when the VP3-genome complex is present in the nucleus, VP1 can be found to colocalize with this complex (Figure 3C, top row). However, there is also a separate pool of VP1 in the nucleus that does not associate with the genome (Figure 3C, bottom row). We now better clarified this point in the manuscript (lines 193, 270 and 274).

2. The disassembly data in 5G- genome was detected but what’s responsible for the change in mobility through the sucrose gradient? Dissociation of VP2 or VP3 from genome? Loss of VP1 pentamers? Loss of histones? Although some blots could clarify the speculation/insight provided in the discussion, this is merely a suggestion to potentially improve the paper- I would not consider this a mandatory requirement for acceptance as it can be addressed in future studies.

For each experiment, we used a fraction of the sample for PCR analysis and subjected the rest of the sample to western blotting in order to address this question. However, given the low level of virus that reaches the nucleus, it was difficult to reliably detect any signal by western. We have added additional discussion to the text (lines 429 and 434) and agree that this is an interesting question that warrants future study.

3. Nup188 silencing resulted in the strongest inhibition of SV40 infection, but other Nups were close. Do these coIP with the Nestrin-2/SV40 comlex too? Does Nestrin-2 silencing affect the interaction between these Nups and SV40? Again, this could be explored in future work.

This is a great question, but we have not tried those experiments. NUP188 is in the inner pore of the NPC so we suspect that Nesprin-2 interacts indirectly with NUP188 and that our IP data likely suggest a more general association between Nesprin-2 and the NPC complex as a whole. Again, we agree that it would be interesting to explore this idea further in future studies to better understand the role of the individual NUPs in viral translocation.

4. What component of SV40 actually binds Nestrin-2? VP3? VP1? If this could be easily clarified it would strengthen the paper, if not then can be addressed in future work.

Although our IP experiments in Figure 2A/2B show that VP1, VP3 (and a low level of VP2) associate with Nesprin-2, this experiment does not reveal the specific SV40 component that binds to Nesprin-2. Our future work will focus on purifying VP1 or VP3 (not a trivial task), and testing if any of these viral components can bind directly to Nesprin-2.

5. The final model figure could be improved to show more details concerning VP1, VP2, VP3, etc. What is the teal aura around the viral genome represent? VP1 pentamers as a capsid?

We have updated the model with more detail showing the likely fate of VP2. We also clarified that the teal aura represents the viral capsid and included a new inset in the figure that explains the organization of VP1, VP2 and VP3 within the viral particle (line 615).

6. The EdU-labeling methodology of SV40 needs to be better detailed in Materials and Methods. What concentration of EdU was used, when was it added post-infection, etc.

We added the EdU for incorporation into the viral particle during virus propagation, but no additional EdU was added during or post-infection. As requested, we have added more details on EdU labeling to the manuscript to clarify (line 639).
